# Environmentally sustainable route to SiO_2_@Au–Ag nanocomposites for biomedical and catalytic applications†

**DOI:** 10.1039/c8ra04502j

**Published:** 2018-09-05

**Authors:** Kanti Sapkota, Prerna Chaudhary, Sung Soo Han

**Affiliations:** School of Chemical Engineering, Yeungnam University 280 Daehak-Ro Gyeongsan Gyeongbuk 38541 Republic of Korea; Department of Nano, Medical & Polymer Materials, College of Engineering, Yeungnam University 280 Daehak-Ro Gyeongsan Gyeongbuk 38541 Republic of Korea sshan@yu.ac.kr +82-53-810-4686 +82-53-810-2773

## Abstract

A facile, sustainable, operationally simple and mild method for the synthesis of SiO_2_@Au–Ag nanocomposites (NCs) using *Nephrolepis cordifolia* tuber extract is described and its catalytic, antibacterial and cytotoxic properties were investigated. The fabricated SiO_2_@Au–Ag NCs were well characterized by UV-visible spectroscopy, transmission electron microscopy (TEM), energy-dispersive X-ray (EDX), Fourier transform infrared (FT-IR) spectroscopy, powder X-ray diffraction (XRD), thermogravimetric analysis (TGA) and X-ray photoelectron spectroscopy (XPS) to determine the optical activity, size and morphology, elemental composition, functional groups present, crystallinity, thermal stability and chemical state respectively. The obtained SiO_2_@Au–Ag NCs exhibited spherical shape SiO_2_ decorated with Au and Ag nanoparticles. The diameter of the SiO_2_ nanoparticles ranges from 200–246 with average 3 nm diameter of Au and Ag NPs. Synthetic utility of this protocol has been demonstrated by exploring its effective catalytic activities for the solvent-free amidation of carboxylic acid with a primary amine with excellent yields. Moreover, the synthesized nanocomposite exhibited as noticeable antibacterial effect against Gram negative and Gram positive bacteria and better bio-compatibility against human keratinocytes. Thus, additive free SiO_2_@Au–Ag NCs display the potential for catalysis and biomedical applications.

## Abstract 2

A facile, sustainable, operationally simple and mild method for the synthesis of SiO_2_@Au–Ag nanocomposites (NCs) using *Nephrolepis cordifolia* tuber extract is described and its catalytic, antibacterial and cytotoxic properties were investigated.

## Graphical abstract

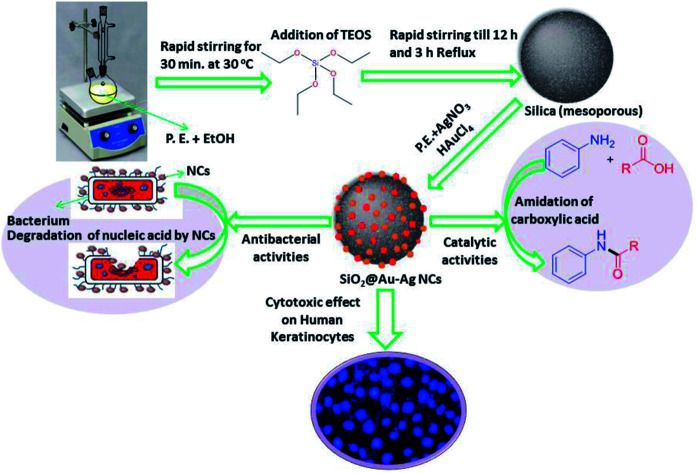

## Introduction

Recent advances in the synthesis of novel nanostructures with metal nanoparticles (Ag, Au, Fe, Cu, Ru, Pd, *etc.*) deposited on metallic oxide surfaces have gained substantial interest in nanotechnology and material science because of their significant applications in diverse fields such as biomedical, catalysis, bio-sensing, information storage, solar cells, optical and many more.^1–7^ Particularly important are silica-metal nanocomposites because of their broad applications in catalysis and biomedical fields.^8,9^ For example, silica based nanocomposites like SiO_2_–Au, SiO_2_–Ag and SiO_2_–Au/Ag display noteworthy advantages in various fields of nanomedicine such as bioimaging, photothermal therapy, drug delivery, biosensing and catalysis due to their highly attractive properties such as tunable porous structure, remarkable plasmonic properties, unique composition, biocompatibility and physiochemical properties.^10–12^ Therefore, the silica-metal nanostructure is a widely used material due to its unique properties like optical transparency, porous structure, ability to functionalize pore system, easy surface modification, biocompatibility and chemical stability.^13–17^ In addition, nanostructured Au and Ag materials are highly applicable in interfacial science and (bio)analytical chemistry because their optical properties are appropriate for applications of surface-sensitive techniques like surface enhanced Raman (SER) spectroscopy.^18–25^ Moreover, over the past few years, silver and gold nanoparticles have been increasingly used due to their powerful optical, electrical and microbial properties in various areas of research as biological sensors, catalysis, drug delivery vehicles, and anti-microbial agents as well as having low cytotoxic effects on mammalian cells.^26–31^

Due to the wide applications and usefulness in various fields, silica-metal nanocomposites have been synthesized by various research groups. For example, β-cyclodextrin (β-CD) coated SiO_2_@Au@Ag nanoparticles has been synthesized in a single-step reaction using β-CD as both the reducing and stabilizing agents for the surface-enhanced Raman scattering (SERS) detection of polychlorinated biphenyls (PCBs).^32^ Recently, silica coated Au/Ag nanomaterials have been reported for photothermal and photodynamic cancer therapy.^33^ Moreover, Au, Ag, Pd/SiO_2_ mesoporous nanocomposites has been synthesized by sonochemical approach.^34^ Monodisperse α-Fe_2_O_3_@SiO_2_@Au core/shell nanocomposite spheres has been synthesized by sol–gel process based on the modified Stöber method.^35^ Gold/silver bi-metal nanodots on silica spheres prepared by *N*-[3-(trimethoxysilyl)propyl]diethylenetriamine and modified strobe method respectively and exhibited antimicrobial properties.^36^ In addition, highly sensitive surface-enhanced Raman scattering (SERS) probes based on SiO_2_@Au@Ag nanoparticles (NPs) and bifunctional Ag@SiO_2_/Au NPs for probing sequential catalytic reactions have been recently reported.^37,38^

Though there are several reports for the synthesis of SiO_2_–metal nanomaterials, most of the reported methods utilize chemical approaches for the synthesis of such nanoparticles. However, these chemical based methods are toxic, hazardous and have side effects for health and environment. Moreover, using toxic and harmful chemicals for the fabrication of metal nanoparticles raises the environmental pollution and toxicity levels. Therefore development of environmental friendly, clean and green approach is necessary for the synthesis of metal nanoparticles to minimize the environmental impact and hazardous chemicals.^39–42^ In order to fulfill the need for developing environmentally benign methods, plant materials can be used as green material that can be easily available at low cost, easy to handle, renewable and more importantly non-toxic and environmental friendly for the preparation of number of metal and metal oxide nanoparticles.^43–46^ Very recently, we have reported Au–Ag@AgCl nanocomposites using AgNO_3_ and HAuCl_4_ precursor and the tuber extract of *Nephrolepis cordifolia* as a reducing and stabilizing agents.^47^

**Scheme 1:**
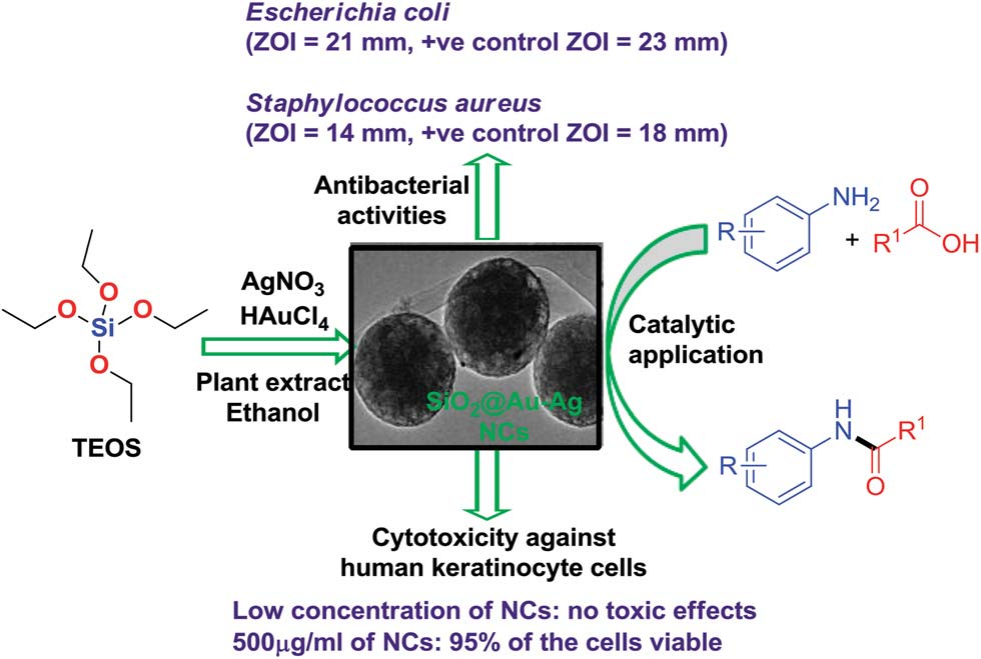
Green synthesis of recyclable SiO_2_@Au–Ag NCs and their diverse catalytic and biomedical applications.

As a continuous effort in developing environmentally benign method for the synthesis of metal nanomaterials, herein, we disclose the synthesis of SiO_2_@Au–Ag nanocomposites through a facile and green technique using tuber extract of *Nephrolepis cordifolia* which acts as both reducing and capping agents without using external additives (Scheme 1). Then, synthesized SiO_2_@Au–Ag NCs were checked for its catalytic potential for the solvent-free amidation of carboxylic acid with primary amine. Furthermore, antibacterial and cytotoxic activities were investigated using the synthesized SiO_2_@Au–Ag NCs.

## Experimental

### Materials and methods

Tetraethyl orthosilicate (TEOS; purity 99.9%), silver nitrate (AgNO_3_, 99%), gold(iii) chloride trihydrate (HAuCl_4_·3H_2_O, 99.9%) and ammonium hydroxide (NH_4_OH) were purchased from Sigma-Aldrich. The fresh tubers of *Nephrolepis cordifolia* (L.) C. Presl was obtained from Kaski district of Nepal. Human keratinocyte cells [CCD 1102 KERTr (ATCC/CRL-2310)] were obtained from the American Type Culture Collection (ATCC). Dulbecco's medium, Phosphate Buffered Saline (PBS), fetal bovine serum (FBS), dimethyl sulphoxide (DMSO) was purchased from Sigma-Aldrich (St Louis, MO, USA). Penicillin, streptomycin, trypsin/EDTA and (4,5-dimethylthiazol-2-yl)-2,5-diphenyltetrazolium bromide (MTT) were also procured from Sigma-Aldrich. The bacterial strains of *Staphylococcus aureus*, (KCTC-1916) and *Escherichia coli* (KCTC-1924) were obtained from Korean Collection for Type Cultures (KCTC). Muller–Hinton broth media for bacterial culture and agar were procured from Becton, Dickinson and Company (Sparks, MD 21152 USA). Firstly, the used glassware was washed and rinsed with double distilled water and then dried in an oven. Double distilled water was used throughout the experiments. All the obtained chemicals were used as received without further purification. The tubers of *Nephrolepis cordifolia* was washed with double distilled water and dried at room temperature. The 5 grams of tubers were cut into small pieces and 100 mL of distilled water was added. Then, the mixture was boiled for 10 minutes. The resultant solution was centrifuged at 4000 rpm for 10 minutes and filtered.

### Synthesis of SiO_2_ NPs

Firstly 100 mL of prepared tuber extract of *Nephrolepis cordifolia* was mixed to 100 mL of ethanol in a 500 mL round bottom flask. Then the mixture was stirred using a magnetic bar for 30 min at 30 °C. After 30 min, 6 mL of tetraethyl orthosilicate (TEOS) added to the extract quickly by dropwise and the pH was adjusted 12.0 using ammonium hydroxide. The resultant mixture was vigorously stirred using a magnetic bar for 18 h and refluxed for 3 h. The presence of white precipitate indicated the formation of silica nanoparticles. Then the reaction was stopped and washed the solution with ethanol 4 times by centrifugation at 10 000 rpm, at 4 °C for 20 min (using a LaboGene 1580R centrifuge: Gyrozen). Finally SiO_2_ nanoparticles was dried in a vacuum oven at 60 °C for 3 h and collected in a sterile airtight vial.

### Synthesis of SiO_2_@Au–Ag nanocomposites (NCs)

The prepared 100 mg of SiO_2_ nanoparticles (NPs) were dispersed in 100 mL double distilled water and sonicated for 1 h. Then, 10 mL of freshly prepared *Nephrolepis cordifolia* tuber extract solution was mixed with 100 mL of 2 mmol AgNO_3_ aqueous solution and mixed with dispersed SiO_2_ NPs solution and sonicated for 30 min. Subsequently, 100 mL of aqueous solution of 2 mmol HAuClO_4_ and 10 mL of tuber extract was added to the dispersed solution. The resultant mixture of solutions was sonicated for 1 h. Then the brownish red color solution indicated the formation of SiO_2_@Au–Ag nanocomposites (NCs). The prepared NCs were washed and isolated by centrifugation at 10 000 rpm, at 4 °C for 20 min and the process was repeated four times and dried in a vacuum oven at 60 °C for 5 h.

### Characterization of NCs

The surface plasmon resonance was performed in order to identify the formation of nanocomposites using a quartz cuvette by a UV-vis spectrophotometer (Shimadzu UV-2600). The powder X-ray diffraction (XRD) was recorded to confirm the crystallinity of the SiO_2_@Au–Ag NCs using a PANalytical X'Pert PRO MPD unit operating at voltage 40 kV and 30 mA with Cu Kα as the X-ray source (*λ* = 1.5406 Å) with scanning 2*θ* angle range, 20–90°, at rate of 1.2° min^−1^. Fourier transform infrared (FT-IR) spectra were recorded using a Perkin-Elmer FT-IR spectrometer in transmittance mode between the wavenumber range 400–4000 cm^−1^. For transmission electron microscopy (FE-TEM) sample was prepared by dipping the copper grid twice into the diluted and 2 h sonicated homogenised NCs sample solution. Then air dried grid was analysed to identify the size and shape of the synthesized NCs using field emission transmission electron microscopy (FE-TEM, FEI Tecnai F20). Similarly, the morphology of the synthesized NCs was characterized by field emission scanning electron microscopy (Fe-SEM, S4800, Hitachi, Ltd.) with an acceleration voltage of 5.0 kV. The elemental analysis of prepared NCs was performed using high-angle annular dark-field scanning TEM energy-dispersive X-ray spectroscopy (HAADF-STEM-EDS) at an accelerating voltage of 200 kV with a point resolution of 0.24 nm, a Cs and Cc of 1.2 mm each with a focal length of 1.7 mm, and by a Genesis liquid nitrogen cooled EDS detector.

X-ray photoelectron spectroscopy (XPS) was carried out to determine the surface chemistry and electronic states of prepared NCs using a Thermo Scientific K-Alpha system an Al K-alpha X-ray source with an ion source energy region of 100 V to 3 keV. Zeta potential of NCs was recorded using Zetasizer Nano ZS (Malvern Instrument). The sample was prepared in distilled water and sonicated for 30 min. The prepared sample was equilibrated for 2 min in a disposable cell and the measurement was conducted three times at 25 °C. Thermogravimetric analyses (TGA) was carried out by observing the percentage of mass loss using a differential scanning calorimeter (TG-DTA, SDT-Q600 V20.5 Build 15) in the temperature range 30 to 800 °C at a heating rate of 10 °C min^−1^ in N_2_ atmosphere.

### Catalytic activity

The synthesized SiO_2_@Au–Ag NCs were used as excellent catalyst for the solvent-free amidation of carboxylic acid with primary amine.

### General procedure for the solvent-free syntheses of amide derivatives 3a–3d

2 mol% SiO_2_@Au–Ag NCs were added to a stirred solution of amine 1 (1.0 mmol) and carboxylic acid 2 (5.0 mmol). The reaction mixture was heated at 100 °C for 8 h and the progress of the reaction was monitored by TLC. After reaction completion, SiO_2_@Au–Ag NCs were recovered *via* hot filtration as residue and the filtrate was subjected to silica gel column chromatography to isolate the pure products 3a–3d.

### Antibacterial activity of SiO_2_@Au–Ag NCs

The antibacterial activities of the green synthesized nanocomposites were evaluated against Gram-positive (*S. aureus* KCTC-1916) as well as Gram-negative (*E. coli* KCTC-1924) bacterium using a modified disc diffusion method.^48^ Briefly, 3–4 colonies of each bacterium were inoculated in 10 mL of Mueller–Hinton (M–H) broth medium and the bacteria were grown at 37 °C in a shaking incubator at 200 rpm until the optical density (O.D) was reached 0.8 at 600 nm. From this bacterial suspension, 100 μl was spread over M–H agar plates. Various concentrations of nanoparticles (50–200 μg mL^−1^) were added to the sterilized 6 mm filter discs (Whatman, GE Healthcare UK Limited) placed on the M–H agar plates. Ampicillin (200 mg L^−1^) was used as reference positive control for the experiments. All the plates were incubated at 37 °C for 24 h and the diameters of zone of inhibition were measured. All the experiments were performed in triplicates and mean diameter was calculated.

### *In vitro* cytotoxicity assay

The cytotoxic effect of SiO_2,_ SiO_2_@Au, SiO_2_@Ag and SiO_2_@Au–Ag nanocomposites was analyzed using a standard MTT (3-(4,5-dimethylthiazol-2-yl)-2,5-diphenyltetrazolium bromide) assay. Human keratinocyte cells were used to assess the cytotoxic effects of the nanoparticles. Briefly, keratinocyte cells [CCD 1102 KERTr (ATCC/CRL-2310)] were grown in Dulbecco's modified Eagle's (DMEM) medium supplemented with 10% fetal bovine serum along with 100 U mL^−1^ penicillin–streptomycin in a humidified environment containing 5% CO_2_/95% air at 37 °C. After reaching the confluence, cells were passaged and seeded in a 96 well plate at a density of 1 × 10^5^ cells per well for overnight. Next day, the cells were treated with various concentrations (50–500 μg mL^−1^) of nanoparticles in triplicates and kept for 24–96 h at 37 °C in a humidified environment. Untreated cells were used as a control.

After treatment, old media was removed and each well was washed with PBS followed by incubation with 50 μl of MTT (5 mg mL^−1^ stock) for 4 h. The purple colored formazan crystal formed, were dissolved in 200 μl DMSO. The absorbance was taken at 570 nm using a multi well ELISA plate reader (BioTek Instruments, Inc., USA). The cell viability were measured using the formula-Cell viability = (O.D of experiment/O.D. of control) × 100

All the readings were taken in triplicates and a graph showing percentage cell viability was plotted against nanoparticles concentration.

## Results and discussion

### Characterization of SiO_2_@Au–Ag NCs

**Fig. 1:**
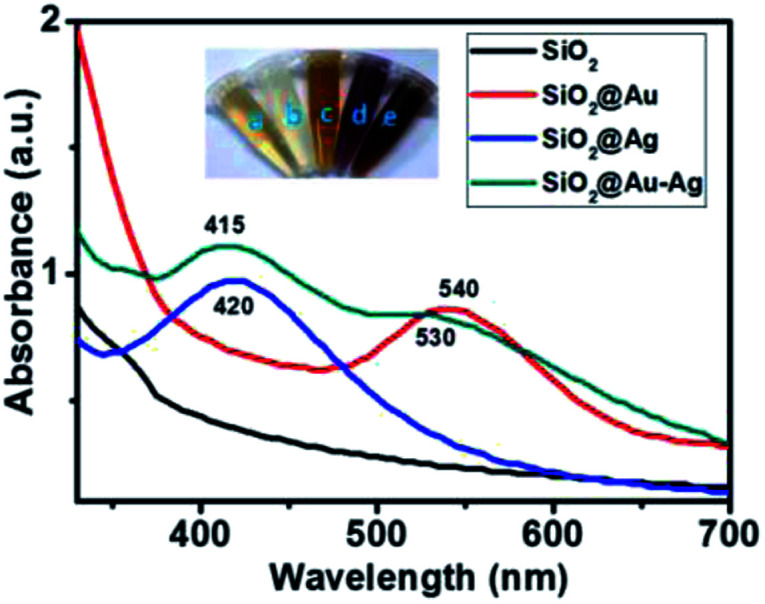
UV-vis spectra of SiO_2_, SiO_2_@Ag, SiO_2_@Au and SiO_2_@Au–Ag. The inset images show: (a) plant extract, (b) SiO_2_, (c) SiO_2_@Ag, (d) SiO_2_@Au and (e) SiO_2_@Au–Ag NCs.

Firstly, formation of brownish red color resultant solution indicated the formation of SiO_2_@Au–Ag nanocomposites. Fig. 1 illustrates the UV spectra of SiO_2_, SiO_2_@Ag, SiO_2_@Au and SiO_2_@Au–Ag nanocomposites. The prepared SiO_2_@Au–Ag nanocomposites showed the characteristic surface plasmon resonance (SPR) bands at 415 nm and 530 nm which can be attributed to the SPR of Au–Ag formed on the surface of SiO_2_ where SiO_2_@Au NCs was observed at 540 nm. The strong absorption band of SiO_2_@Ag NCs was observed at 420 nm which ascribed to the formation of Ag on the surface of SiO_2_. The observed characteristic SPR peaks and color change of the NCs indicated the formation of SiO_2_@Au–Ag nanocomposites.

**Fig. 2:**
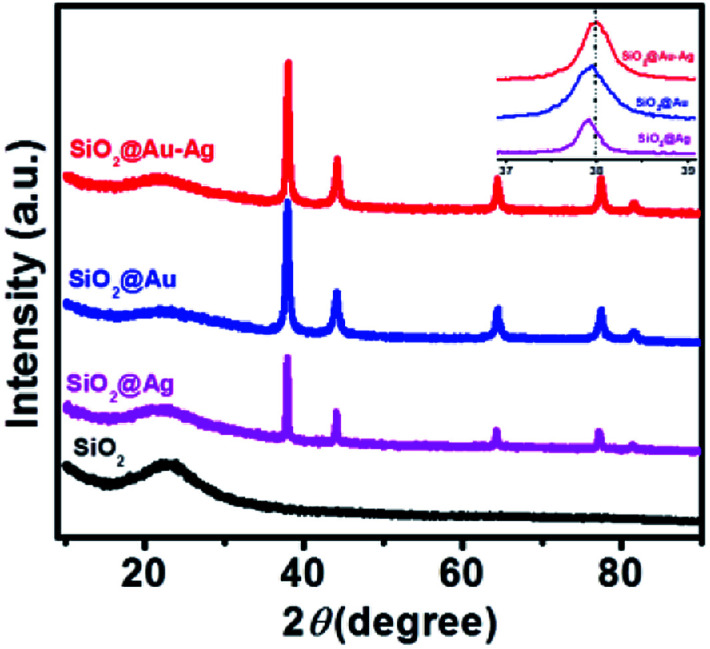
XRD patterns of the SiO_2_, SiO_2_@Ag, SiO_2_@Au and SiO_2_@Au–Ag NCs. Inset shows the magnified view of SiO_2_@Au–Ag, SiO_2_@Au and SiO_2_@Ag.

Fig. 2 shows the powder X-ray diffraction pattern of SiO_2_@Au–Ag synthesized from *Nephrolepis cordifolia* with intense characteristic peaks. In Fig. 2, the XRD peaks of *Nephrolepis cordifolia* mediated SiO_2_@Au–Ag observed at 2*θ* value of 38.2°, 44.4°, 64.6°, 77.6° and 81.7° were indexed to the reflection planes (111), (200), (220), (311), and (222) respectively, due to the face-centred cubic phase of silver (JCPDS no. 04-0784) and cubic gold (JCPDS no. 04-0783), which confirmed the crystalline nature of Ag and Au metal species, whereas SiO_2_ NPs didn't show any phase pattern because of amorphous in phase. The diffraction pattern of the silica nanoparticles were studied around 21° at 2*θ* value.

**Fig. 3:**
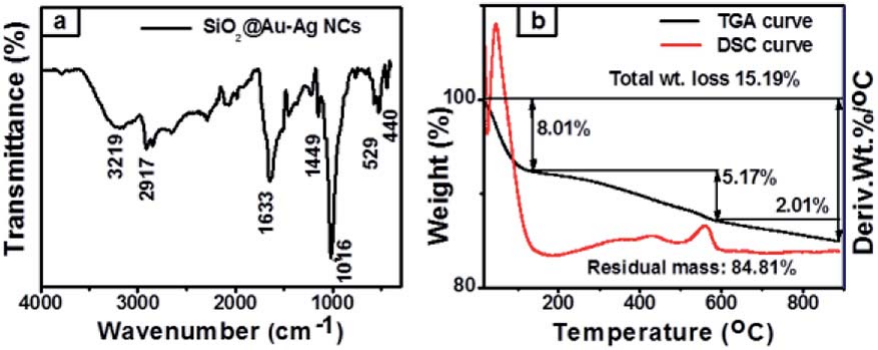
(a) FT-IR spectra and (b) TGA and DSC curve of SiO_2_@Au–Ag NCs.

Fig. 3a shows the FT-IR spectra of synthesized nanocomposites which analyze functional groups present in the *Nephrolepis cordifolia* mediated SiO_2_@Au–Ag NCs as both capping agents as well as reducing agents which are responsible for the fabrication of nanocomposites. As presented in Fig. 3a, the absorption band at 3219 cm^−1^ of obtained SiO_2_@Au–Ag NCs corresponds to O–H stretching of alcohols or phenols and aromatic C–H stretching. The absorption peaks at wave number 1633 cm^−1^ assigned to asymmetric stretching of COO^−^ and 1449 cm^−1^ are attributed to the symmetric stretching. The FTIR absorption band observed at around 1016 cm^−1^ corresponds to asymmetric vibration of Si–O–Si.^49^

**Fig. 4:**
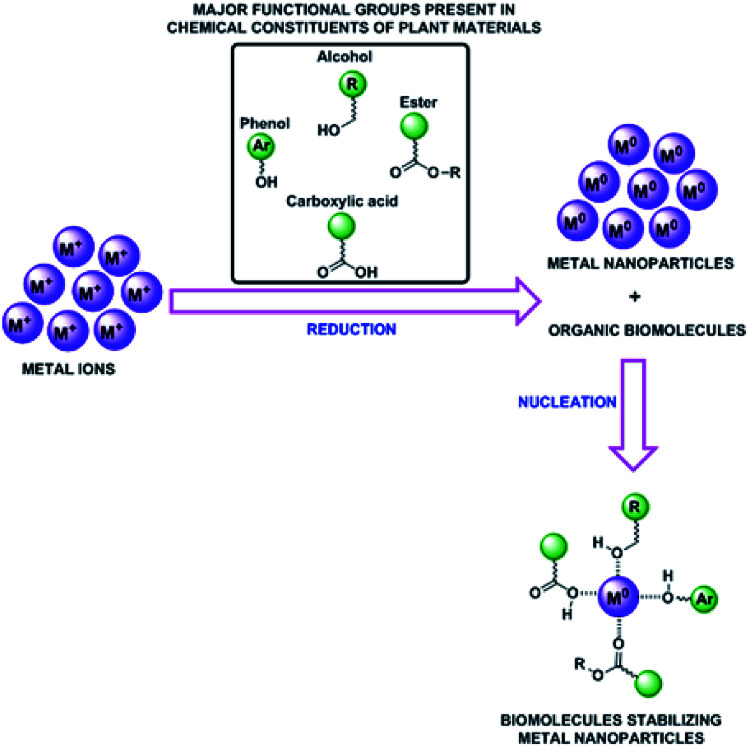
Proposed mechanisms for the green synthesis of SiO_2_@Au–Ag NCs.

A number of bioactive molecules present in the plant materials play a vital role as a reducing and capping agents for the formation of nanocomposites. The reducing phytochemicals which bind and capped the metal ion to form the stable nanoparticles as mechanism presented in Fig. 4. Therefore, the organic molecules which played the main role for formation of SiO_2_@Au–Ag nanocomposites are phytol, ethyl palmitate, β-ionone, ethyl linolenate diterpene alcohol, eugenol, anethol and α-cadinol which have been extracted from *Nephrolepis cordifolia*.^50^

Thermal stability of SiO_2_@Au–Ag NCs prepared from tuber extract of *Nephrolepis cordifolia* was examined by DSC-TGA as illustrated in Fig. 3b. The sample of prepared NCs was subjected at temperature starting from room temperature to 900 °C with ramp rate 10 °C min^−1^ under nitrogen atmosphere. The decomposition of mass was recorded in three successive steps. The first step of mass loss was found to be 8.01% around temperature 100 °C due to the moisture and some volatile compounds contains in the sample. Secondly, 5.17% and finally 2.1% mass loss at the decomposition temperature 430 °C and beyond 900 °C were examined respectively due to the loss of capping agents and other bioactive molecule present on the *Nephrolepis cordifolia* mediated NCs. Thus, the total percentage of mass degradation (15.19%) was observed in TGA graph. The remaining percentage of mass (84.81%) of sample indicated the existence of Si, O, Au and Ag in biosynthesized SiO_2_@Au–Ag NCs.

**Fig. 5:**
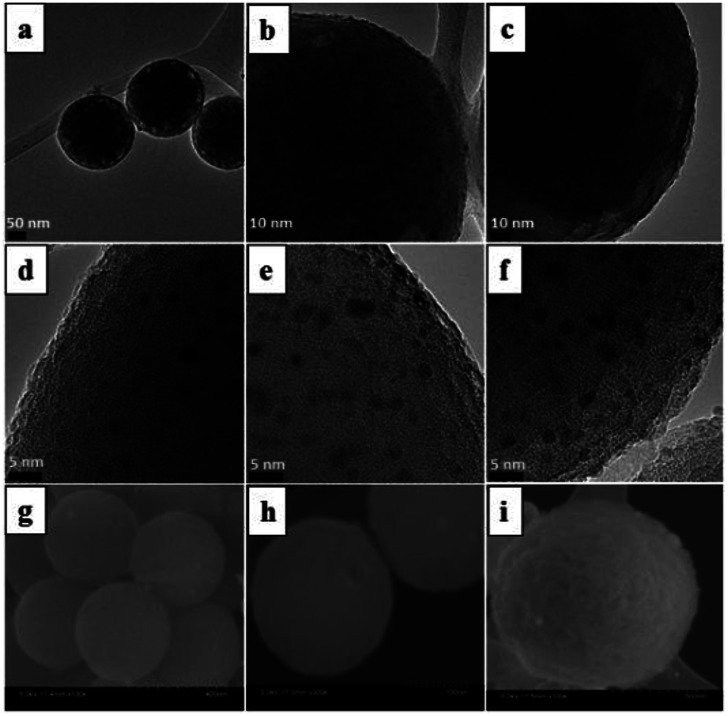
TEM images of SiO_2_@Au–Ag NCs at scale bar (a) 50 nm, (b) 10 nm (c) 10 nm (d) 5 nm (e) 5 nm (f) 5 nm and SEM images at (g) 400 nm (h) 100 nm and (i) 500 nm.

**Fig. 6:**
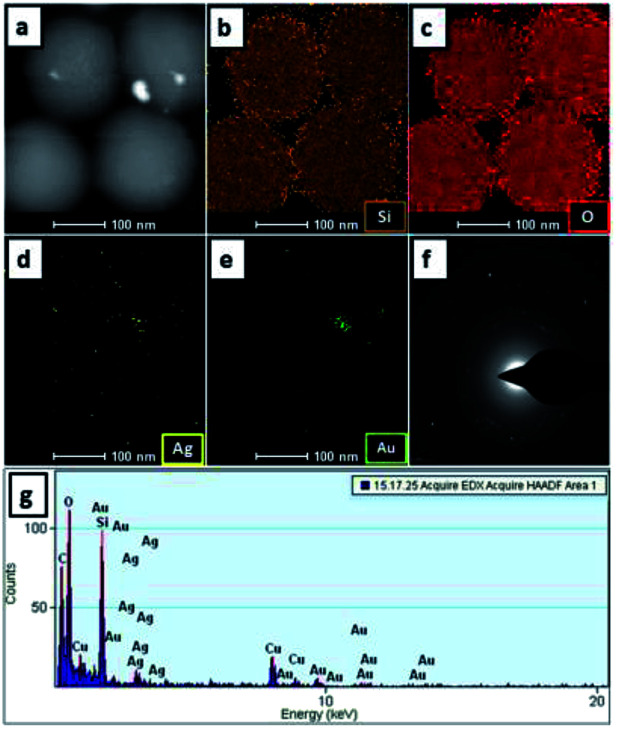
HAADF-STEM micrographs at 100 nm (a–e), (f) SAED pattern and (g) EDS elemental mapping of Si, O, Au and Ag in SiO_2_@Au–Ag NCs.

TEM and SEM analysis revealed the morphology and size of the SiO_2_@Au–Ag NCs prepared from *Nephrolepis cordifolia* tuber (Fig. 5). Fig. 5a–f displays TEM images and HR-TEM of SiO_2_@Au–Ag NCs which confirmed the spherical shape of Au and Ag nanoparticles decorated on the porous spherical shape silica nanoparticles. TEM micrographs investigated the polydispersed silica nanoparticles with size ranges 200–246 nm. HRTEM image clearly showed the well dispersed silver–gold nano particles on the porous silica nanoparticles (Fig. 5b–f). Similarly, SEM analysis displayed the spherical morphology of the synthesized NCs and the Au and Ag NPs could not be clearly visible on the surface of SiO_2_ due to the very small size of Au and Ag NPs (3 nm) in low resolution SEM (Fig. 5g–i). The formation of SiO_2_@Au–Ag system could be due to the strong interaction of Au–Ag metals with the support material SiO_2_. In general, SiO_2_ is regarded as an inert support and does not show significant metal support interaction (SMSI). However, in Pt–SiO_2_ system, SMSI effect has been demonstrated.^51^ Moreover, a covalent interaction has been reported between metal atoms and surface anions in metal–SiO_2_ system.^52,53^Fig. 6a–e display HAADF-STEM images of Si, O, Au and Ag present in the SiO_2_@Au–Ag NCs using the high-angle annular dark-field imaging with scanning transmission electron microscopy (HAADF-STEM). Fig. 6f shows the SAED pattern of the SiO_2_@Au–Ag NCs. Furthermore, energy-dispersive X-ray spectroscopy (EDS) analysis revealed the elemental composition of synthesized nanocomposites which confirmed the presence of Si, O, Au and Ag element in the SiO_2_Au@Ag NCs (Fig. 6g).

**Fig. 7:**
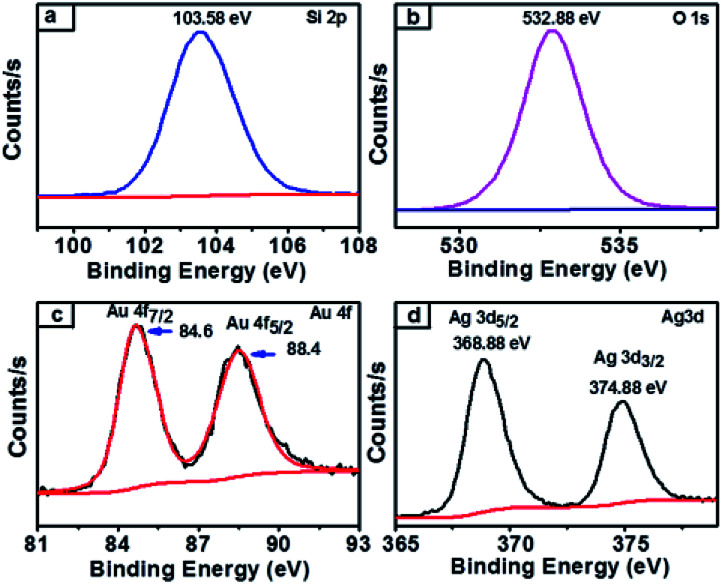
XPS spectra of SiO_2_@Au–Ag NCs (a) Si 2p-electrons of silica containing NCs (b) O 1s-electrons of oxygen containing NCs (c) Au 4f-electrons of gold containing NCs and (d) Ag 3d-electrons of silver containing NCs.

XPS survey spectra of synthesized nanocomposites which confirmed the chemical state of the NCs and further clarified the silica, gold and silver along with carbon elements present in the obtained SiO_2_@Au–Ag NCs (Fig. S1, ESI†). The spectrum of carbon element present in the figure indicated the presence organic compound in plant mediated SiO_2_@Au–Ag NCs. As presented in Fig. 7a, the single Si 2p spectrum at 103.5 eV attributed to the binding energy of Si in SiO_2_.^35^ Similarly, the XPS Au 4f spectra displayed the double bands at 84.6 eV and 88.4 eV binding energies for Au 4f_7/2_ and Au 4f_5/2_ respectively in agreement with the characteristic binding energy of Au^0^ (Fig. 7c).^54^ In Fig. 7d, Ag 4d XPS band showed two peaks at the binding energies of 368.8 eV and 374.8 eV which belong to Ag 3d_5/2_ and Ag 3d_3/2_ along with spin splitting of 6.0 eV respectively which confirmed the presence of Ag^0^.^55,56^ From the XPS analysis, the surface atomic percentage of Si, O, Au and Ag was found to be 30.07%, 68.49%, 0.22% and 1.21% respectively.

### Stability analysis of SiO_2_@Au–Ag

**Fig. 8:**
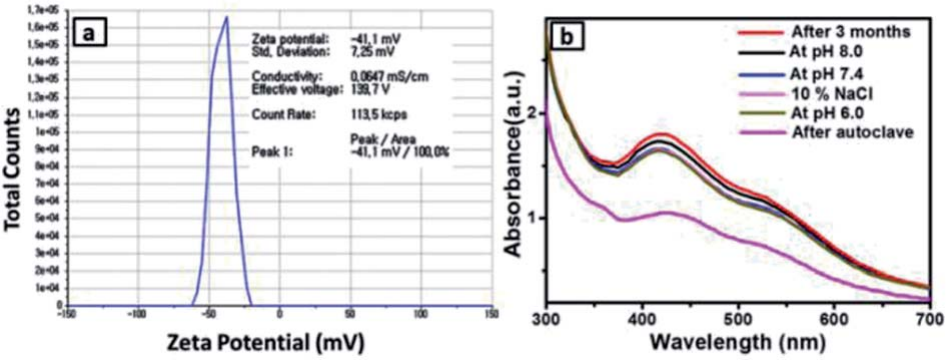
(a) Zeta potential of the NCs. and (b) UV-vis spectra in various conditions (after 3 months, pH 6.0, pH 7.4, pH 8.0, 10% NaCl and after autoclave).

The stability of green synthesized SiO_2_@Au–Ag NCs using *Nephrolepis cordifolia* was evaluated as shown in Fig. 8. Firstly, the prepared NCs was analysed by observing zeta potential distribution and the value was obtained 41.1 mV. The higher negative zeta potential value of *Nephrolepis cordifolia* stabilized SiO_2_@Au–Ag NCs confirmed the formation of stable NCs (Fig. 8a).^47,57^ Furthermore, the surface plasmon resonance (SPR) spectra of prepared SiO_2_@Au–Ag NCs was investigated using spectrophotometry in various conditions such as in different pH conditions (at pH 8, 7.4, and 6) along with using 10% NaCl and after autoclave the sample at 121 °C for 60 min. The Fig. 8b displays the SPR peaks which showed nearly same wave length (∼425 nm) in every condition, which further confirmed the stability of green synthesized SiO_2_@Au–Ag NCs in biological fluids.

### Catalytic activity

The optimization of reaction condition for the solvent free amidation of carboxylic acid is presented in Table 1. The highest yield of the product 3a was obtained using SiO_2_@Au–Ag NCs compared to other catalysts such as SiO_2_, SiO_2_@Ag, SiO_2_@Au. In absence of catalyst, the yield was even lower. Based on the optimization study, catalytic activities of the synthesized SiO_2_@Au–Ag NCs for the solvent-free amidation of carboxylic acid with primary amine is summarized in Table 2. Reaction of aniline (1a) with acetic acid (2a) in the presence 2 mol% SiO_2_@Au–Ag NCs at 100 °C for 8 h provided amide 3a in 97% yield (entry 1, Table 2). Similarly, reaction between 4-methoxy aniline (1b) or 2-chloro aniline (1c) and aniline (2a) with acetic acid (2a) in the presence 2 mol% SiO_2_@Au–Ag NCs at 100 °C for 8 h provided the desired products 3b and 3c in 98% and 95% yields respectively (entry 2 and 3, Table 2). Moreover, treatment of aniline (1a) with trifluoroacetic acid (2b) in the presence 2 mol% SiO_2_@Au–Ag NCs at 100 °C for 8 h provided amide 3d in 95% yield (entry 4, Table 2). These results demonstrate the efficient catalytic activities of the synthesized SiO_2_@Au–Ag NCs for the solvent-free amidation of carboxylic acid with primary amine in good yield. The structure of compounds 3a–3d was identified by ^1^H and ^13^C NMR and by direct comparison with reported data (data and spectra of the synthesized compounds are presented in the ESI†).

**Table 1:** Optimization of the reaction conditions for the synthesis of 3a^a^

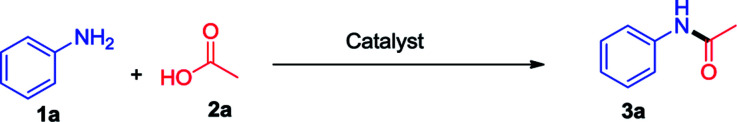			
Entry	Catalyst (2 mol%)	Conditions	Yield^b^ (%)
1	SiO_2_	8 h, 100 °C	56
2	SiO_2_@Ag	8 h, 100 °C	65
3	SiO_2_@Au	8 h, 100 °C	67
**4**	**SiO**_**2**_ **@Au–Ag**	**8 h, 100 °C**	**97**
5	—	8 h, 100 °C	32

a Reaction conditions: amine 1a (1.0 mmol), carboxylic acid 2a (5 mmol).

b Isolated yield.

**Table 2:** SiO_2_@Au–Ag NCs catalyzed solvent free amidation of carboxylic acid with primary amine^a^

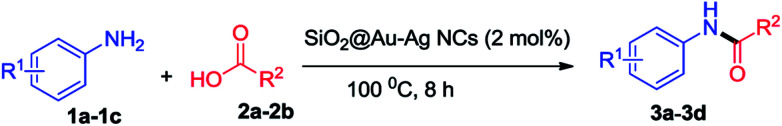
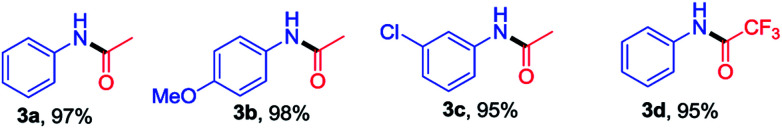

a Reaction conditions: amine 1 (1.0 mmol), carboxylic acid 2 (5 mmol) and SiO_2_@Au–Ag NCs (2.0 mol%).

### Comparative study of the SiO_2_@Au–Ag NCs with other catalysts

The enhanced catalytic performance of the synthesized SiO_2_@Au–Ag NCs can be evaluated by comparing the observed result with that of other reported catalysts. The results are outlined in Table 3.^58–63^ We compared the results of solvent free amidation of carboxylic acid with primary amine in the presence of various catalysts with respect to the solvent, temperature and product yield. Eventhough, the reported methods have their own advantages, the amidation of carboxylic acid in the presence of the biosynthesized SiO_2_@Au–Ag NCs were carried out rapidly with high yields and low loading of the catalyst.

**Table 3:** Comparative catalytic activity of SiO_2_@Au–Ag with other catalysts

Entry	Catalyst	Temperature	Yield (%)	Ref.
1	Thiamine hydrochloride	100 °C	90	58
2	PANI emarldine	Dioxane, reflux	87	59
3	Nb_2_O_5_	Toluene, reflux	80	60
4	rGO-SO_3_H	Ultrasonic, rt	94	61
5	Yttria-zirconia	125 °C	93	62
6	Starbon-400-SO_3_H	130 °C	87	63
7	SiO_2_@Au–Ag NCs	100 °C	97	Present work

### Antimicrobial activity of SiO2@Au–Ag NCs

**Fig. 9:**
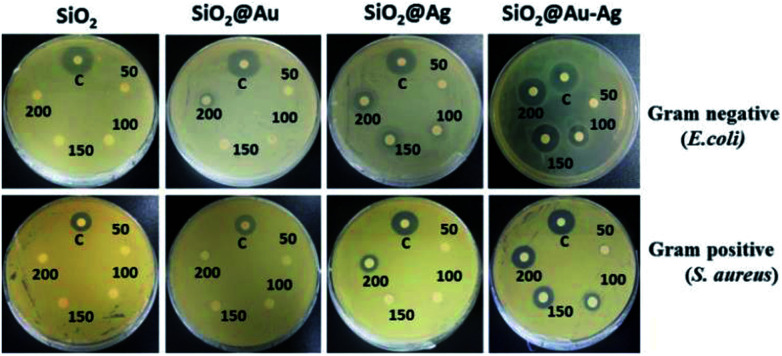
Images of comparative antibacterial studies of different nanoparticles SiO_2_, SiO_2_@Au, SiO_2_@Ag and SiO_2_@Au–Ag at different concentration 50 μg mL^−1^, 100 μg mL^−1^, 150 μg mL^−1^, 200 μg mL^−1^ against bacteria *E. coli* and *S. aureus* with respect to positive control of ampicillin (200 μg mL^−1^).

The antibacterial activity of synthesized nanoparticles was investigated against *Staphylococcus aureus* and *Escherichia coli* bacteria. The bactericidal effects of different nanoparticles as depicted by the zone of inhibition are shown in the Fig. 9. From the results, different patterns of zone of inhibition were observed. As shown in the figure, SiO_2_@Au–Ag nanocomposites were found to be most effective against both Gram-positive as well as Gram-negative bacterium at all the concentrations; on the other hand SiO_2_ nanoparticles were inert for both bacterial species at all the tested particle concentrations (Fig. 9). SiO_2_@Au nanoparticles were also found to be inactive for Gram-positive bacterium at all the concentrations used, whereas for Gram-negative bacterium, only at higher concentration (200 μg mL^−1^), an inhibition zone of 10 mm could be seen (Fig. 9). Although the exact mechanism of antibacterial effect of gold nanoparticles is not fully understood but it is assumed that Au NPs binds to the cell wall of the bacterium and suppressed bacterial growth at the first stage of their reproduction.^64^ Due to their well-developed surface chemistry and the smaller size, gold nanoparticles can interact with the microorganisms with ease and cause destructive effects on them.^65^

SiO_2_@Ag nanoparticles showed better antibacterial effects than the SiO_2_@Au nanoparticles and were able to hinder the growth of both bacterial species. This difference might be due to the strong antibacterial properties of silver. In case of SiO_2_@Ag nanoparticles, the inhibition diameter for *E. coli* was measured 0, 7, 11 and 18 mm at a particle concentration of 50 μg mL^−1^, 100 μg mL^−1^, 150 μg mL^−1^ and 200 μg mL^−1^ respectively, whereas for *S. aureus* it was measured 6.5 and 12 mm at 150 and 200 μg mL^−1^ concentrations respectively, Table 2 (Fig. 9). Silver nanoparticles bind to the thiol groups present on the bacterial membrane and create the pores on the cell membrane. As a result there is a complete damage of the membrane which leads to the death of the bacterium.^66^ In case of SiO_2_@Au–Ag nanoparticles, a clearance zone of 7, 13, 18 and 21 mm was measured against *E. coli* at 50 μg mL^−1^, 100 μg mL^−1^, 150 μg mL^−1^ and 200 μg mL^−1^ concentrations respectively as shown in Table 2. A diameter of 23 mm was measured for positive control ampicillin (200 μg mL^−1^), which was comparable to the nanoparticles concentration. However, zone of inhibition of 7, 10, 13 and 14 mm was observed against Gram positive bacteria *S. aureus* at 50 μg mL^−1^, 100 μg mL^−1^, 150 μg mL^−1^ and 200 μg mL^−1^ concentrations respectively (Table 4 and Fig. 9).

**Table 4:** Antibacterial activity of *Nephrolepis cordifolia* mediated SiO_2_, SiO_2_@Au, SiO_2_@Ag and SiO_2_@Au–Ag NCs using disc diffusion method

	Concentration (mg mL^−1^)	Zone of inhibition (ZOI) in mm				
		SiO_2_	SiO_2_@Au	SiO_2_@Ag	SiO_2_@Au–Ag	
Gram –ve bacteria *E. coli* (KCTC-1924)	50	—	—	—	7	For positive control ampicillin (200 mg mL^−1^) ZOI = 23 mm
	100	—	—	—	13	
	150	—	—	7	18	
	200	—	10	11	21	
Gram +ve bacteria *S. aureus* (KCTC-1916)	50	—	—	—	7	For positive control ampicillin (200 mg mL^−1^) ZOI = 18 mm
	100	—	—	—	10	
	150	—	—	6.5	13	
	200	—	—	12	14	

The highest antibacterial activities displayed by SiO_2_@Au–Ag nanocomposites might be due to the synergistic effect of Au–Ag nanoparticles. When particles are alone, they are likely to be aggregate and their surface gets oxidized, which in turn reduce their antibacterial performances, on the other hand together they tend to be more stable with higher antimicrobial activities.^67^ The Gram-positive bacterium were less sensitive to the nanoparticles in comparison to the Gram-negative bacterium, and only a higher concentrations of nanoparticles could inhibit the growth of Gram-positive bacterium. This might be due to the presence of thick, lipoteichoic acid containing peptidoglycan cell wall around the Gram-positive bacterial membrane, which was difficult to cross by nanoparticles at lower concentrations.^68^

### Analysis of cytotoxic effects of nanocomposites

**Fig. 10:**
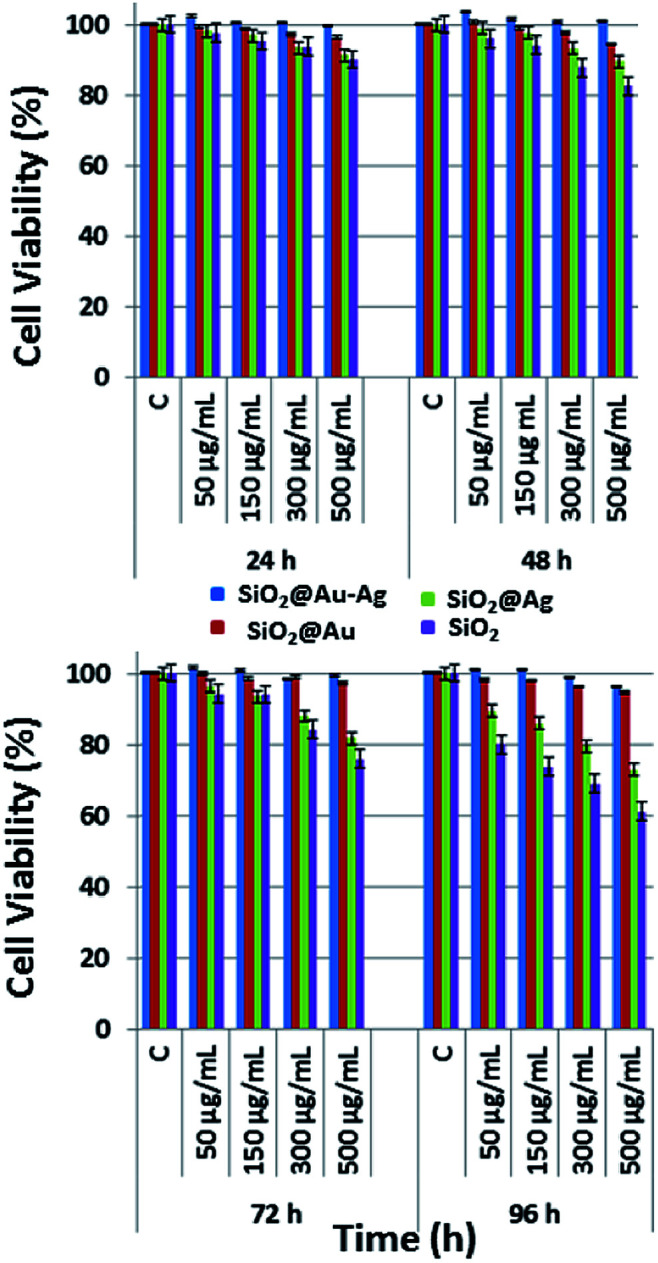
Cytotoxic effect of different nanoparticles SiO_2_, SiO_2_@Au. SiO_2_@Ag and SiO_2_@Au–Ag with different concentrations 50 μg mL^−1^, 150 μg mL^−1^, 300 μg mL^−1^ and 500 μg mL^−1^ on human keratinocytes.

The cytotoxic effect of SiO_2,_ SiO_2_@Au, SiO_2_@Ag and SiO_2_@Au–Ag nanocomposites on human keratinocyte cells was studied using the MTT assay (Fig. 10). MTT assay helps in determination of cell viabilities *in vitro* conditions. The dehydrogenase enzymes present in the metabolically active cells convert yellow colored MTT (3-(4,5-dimethylthiazol-2-yl)-2,5-diphenyl tetrazolium bromide) salt solution to an insoluble violet-blue formazan which can be dissolved in organic solvents like DMSO and analyzed spectrophotometrically. The number of viable cells is directly proportional to the intensity of formazan crystal produced.^69^ The viability of the cells depends on various factors like, concentration, shape, size and incubation period of the nanoparticles.^70^ The results obtained showed concentration dependent cytotoxic effects on keratinocytes after exposure of the cells to the various concentrations (50–500 μg mL^−1^) of nanoparticles. With the increase in the exposure time of nanoparticles from 24–96 hours, cytotoxicity was also increased. As shown in the Fig. 10, SiO_2_ nanoparticles were most toxic to the cells, and in 96 hours at a concentration of 500 μg mL^−1^, almost 40% of the cells were dead. It is assumed that the SiO_2_ nanoparticles interact with the macromolecules of the cells which activate the apoptotic and p53 pathway leading to the generation of reactive oxygen species (ROS) and finally causing the death of the cells.^71^ SiO_2_@Ag nanoparticles also induced cellular toxicity to the keratinocyte cells in a concentration and time dependent manner. After exposure of keratinocyte cells to SiO_2_@Ag nanoparticles at a concentration of 500 μg mL^−1^ for 96 hours, the cell viability was reduced to 75% (Fig. 10). It is supposed that SiO_2_@Ag nanoparticles bind to the cell membranes which disturb membrane permeability by increasing the ionic concentration and the oxidative stress causes the cell death.^72^ SiO_2_@Au nanoparticles were not very much toxic to the keratinocyte cell and longer exposure of the cells with higher concentrations of nanoparticles could exerts their cytotoxic effect (Fig. 10). Conceptually, the cellular uptake of nanoparticles increases with the increase in their concentration and treatment time, which cause higher cytotoxic effect on the cells.^73^ Maximum viability, was observed in the cells which were treated with SiO_2_@Au–Ag nanocomposites. From Fig. 10, it can be seen that only the highest concentration 500 μg mL^−1^ of particle induced the cell death and at this concentration 95% of the cells were viable, however the lower concentrations of nanoparticles did not exert any toxic effect on the cells. The expected reason might be the tight binding of the gold and silver cations on to the surface of silica nanoparticles, which caused a slow release of Ag and Au ions causing higher cell viabilities.^74^ These results demonstrate that the synthesized nanocomposite is safe and biocompatible for potential bio-medical applications.

**Fig. 11:**
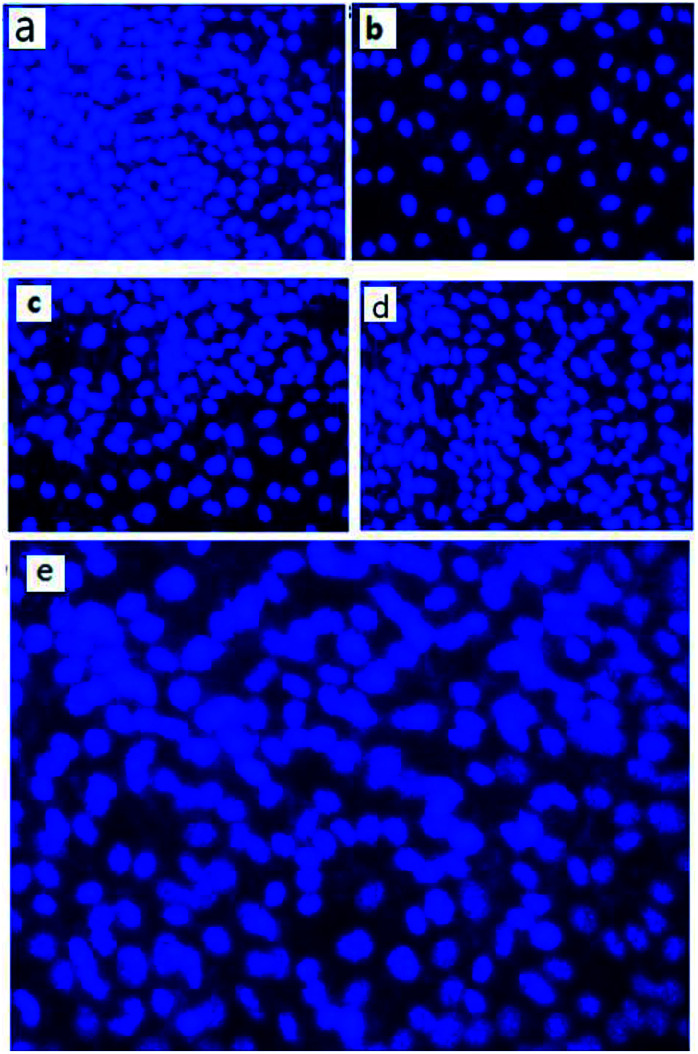
Representative images from independent experiments when human keratinocyte cells were treated with biosynthesized nanoparticles at 500 μg mL^−1^ concentration and cells stained with DAPI after 96 hours, (a) control cells (b) cells treated with SiO_2_ (c) SiO_2_@Ag (d) SiO_2_@Au and (e) SiO_2_@Au–Ag NCs.

The cytotoxic effect of nanoparticles on human keratinocyte cells was further evaluated by staining with DAPI (4′,6-diamidino-2-phenylindole). Briefly, 1 × 10^3^ cells keratinocyte cells were seeded into culture plates and were incubated in a CO_2_ incubator at 37 °C for 24 hours. We can correlate these results with that of MTT assay, where similar kind of effects were observed. Both MTT and cell staining experiments showed a concentration dependent cell death of keratinocyte cells after treatment with the biosynthesized nanoparticles (Fig. 11).

### Recyclability of catalyst

**Fig. 12:**
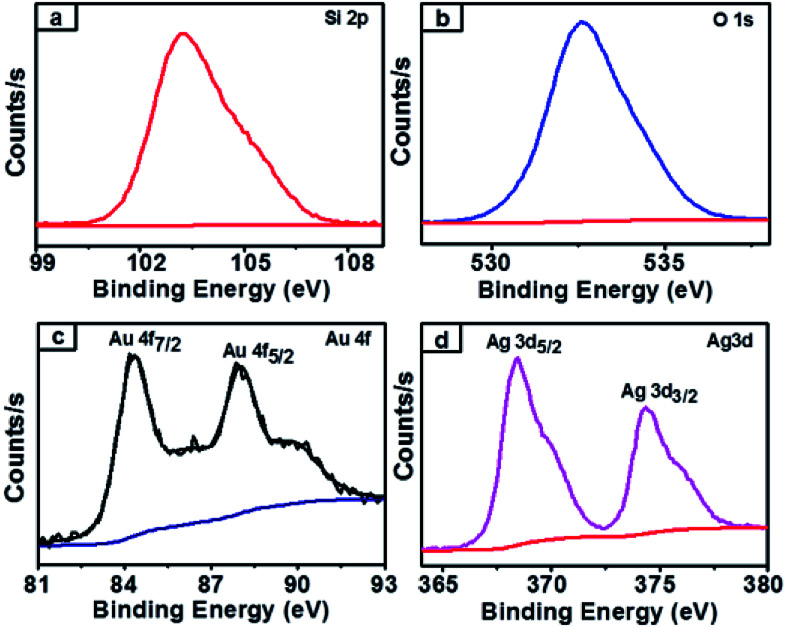
XPS spectra of SiO_2_@Au–Ag NCs after recycle (a) Si 2p-electrons of silica (b) O 1s-electrons of oxygen (c) Au 4f-electrons of gold (d) Ag 3d-electrons of silver of SiO_2_@Au–Ag NCs.

**Fig. 13:**
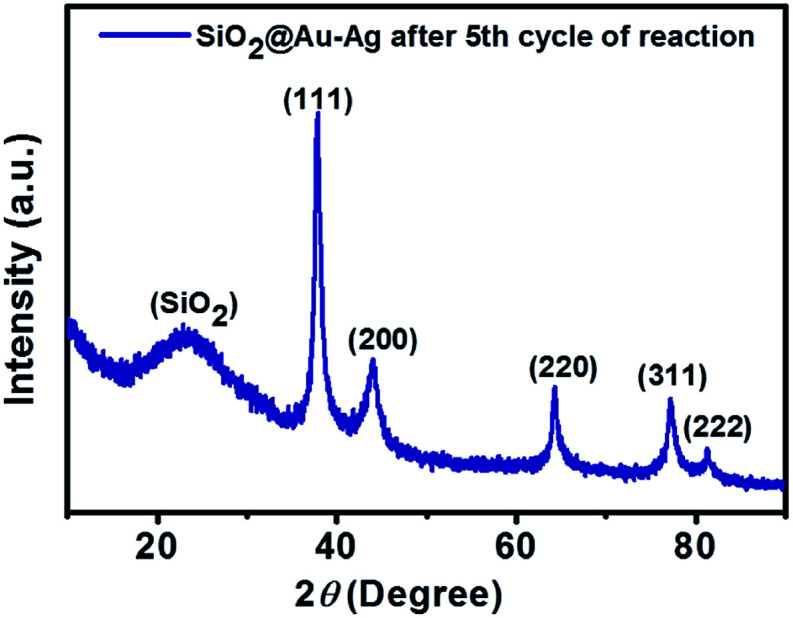
XRD pattern of SiO_2_@Au–Ag NCs after fifth cycle of reaction.

SiO_2_@Au–Ag NCs fabricated by *Nephrolepis cordifolia* was recycled and reprocessed five times without loss of any chemical compositions and catalytic activities (Table 5). After the completion of each reaction, the nanocatalyst was recovered by washing with ethyl acetate followed by hot water filtration for four times. Then the recycled nanocatalyst was dried in a vacuum oven for 4 h at 70 °C. In order to investigate the stability of the recycled nanocatalysts, XRD and XPS analysis was further performed. The XPS and XRD results showed the similar surface chemical compositions and crystallinity of the synthesized SiO_2_@Au–Ag nanocomposites before and after catalysis process without any alterations (Fig. S1, ESI,†Fig. 12 and 13).

**Table 5:** Recyclability of the SiO_2_@Au–Ag NCs^a^

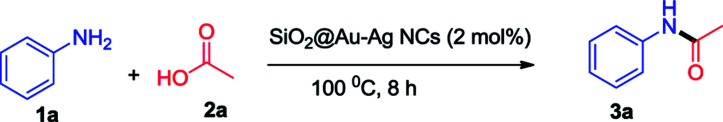		
Entry	Cycle	Yield^b^ (%)
1	First	97
2	Second	95
3	Third	93
4	Fourth	92
5	Fifth	90

a Reaction conditions: amine 1a (1.0 mmol), acid 2a (5.0 mmol), SiO_2_@Au–Ag nanocatalyst (2 mol%).

b Isolated yield.

## Conclusions

An efficient, external additive-free and green approach for the facile synthesis of SiO_2_@Au–Ag NCs using tuber extract of *Nephrolepis cordifolia* was successfully developed. The morphology and size of the synthesized Au/Ag nanocomposites with an average diameter of 3 nm decorated in silica nanoparticles with highly dispersed manner were investigated by transmission electron microscopy (TEM) analysis. The crystallinity and functional group present in the plant mediated NCs were confirmed by X-ray diffraction (XRD) and Fourier transform infrared (FT-IR) analysis. Chemical composition and oxidation state of prepared NCs were confirmed by XPS and EDS analysis. Synthetic functions of fabricated nanocatalyst were established by solvent-free amidation of carboxylic acid with primary amine. In addition, the synthesized nanocomposites demonstrated efficient catalytic activity along with good recyclability. Furthermore, the prepared nanocomposites showed potent bactericidal activities and good biocompatibility. Therefore, the green synthesized SiO_2_@Au–Ag nanocomposites could be used as cost effective, sustainable, additive free, effective catalyst in organic synthesis as well as safe and biocompatible NCs for *in vivo* bio-medical applications.

## Conflicts of interest

The authors declare no competing financial interest.

## Supplementary Material

RA-008-C8RA04502J-s001
