# Supplementary material for: Environmentally sustainable route to SiO2@Au–Ag nanocomposites for biomedical and catalytic applications
Source: RSC Adv. 2018 Sep 5;8(55):31311–21. doi: 10.1039/c8ra04502j (PMC9085631; doi:10.1039/c8ra04502j)
Supplement: RA-008-C8RA04502J-s001 [file RA-008-C8RA04502J-s001.pdf]

Electronic Supplementary Material (ESI) for RSC Advances.
This journal is © The Royal Society of Chemistry 2018

Environmentally sustainable route to SiO_2@Au-Ag nanocomposites for biomedical and catalytic applications

Kanti Sapkota[a,b], Prerna Chaudhary, [a,b] Sung Soo Han[a,b]*

[a]School of Chemical Engineering, Yeungnam University, 280 Daehak-Ro, Gyeongsan, Gyeongbuk 38541, Republic of Korea

[b]Department of Nano, Medical & Polymer Materials, College of Engineering, Yeungnam University, 280 Daehak-Ro, Gyeongsan, Gyeongbuk 38541, Republic of Korea

*Corresponding author

Prof. Sung Soo Han, Email: sshan@yu.ac.kr

Tel: +82–53–810–2773; Fax: +82–53–810–4686

[illegible] catalyzed tandem coupling/hydroarylation

Table of Contents

XPS survey spectra of SiO_2@Au-Ag NCs before and after catalysis **S2**

^{1}H NMR and ^{13}C NMR data and spectra of compounds 3a-3d **S3-S6**

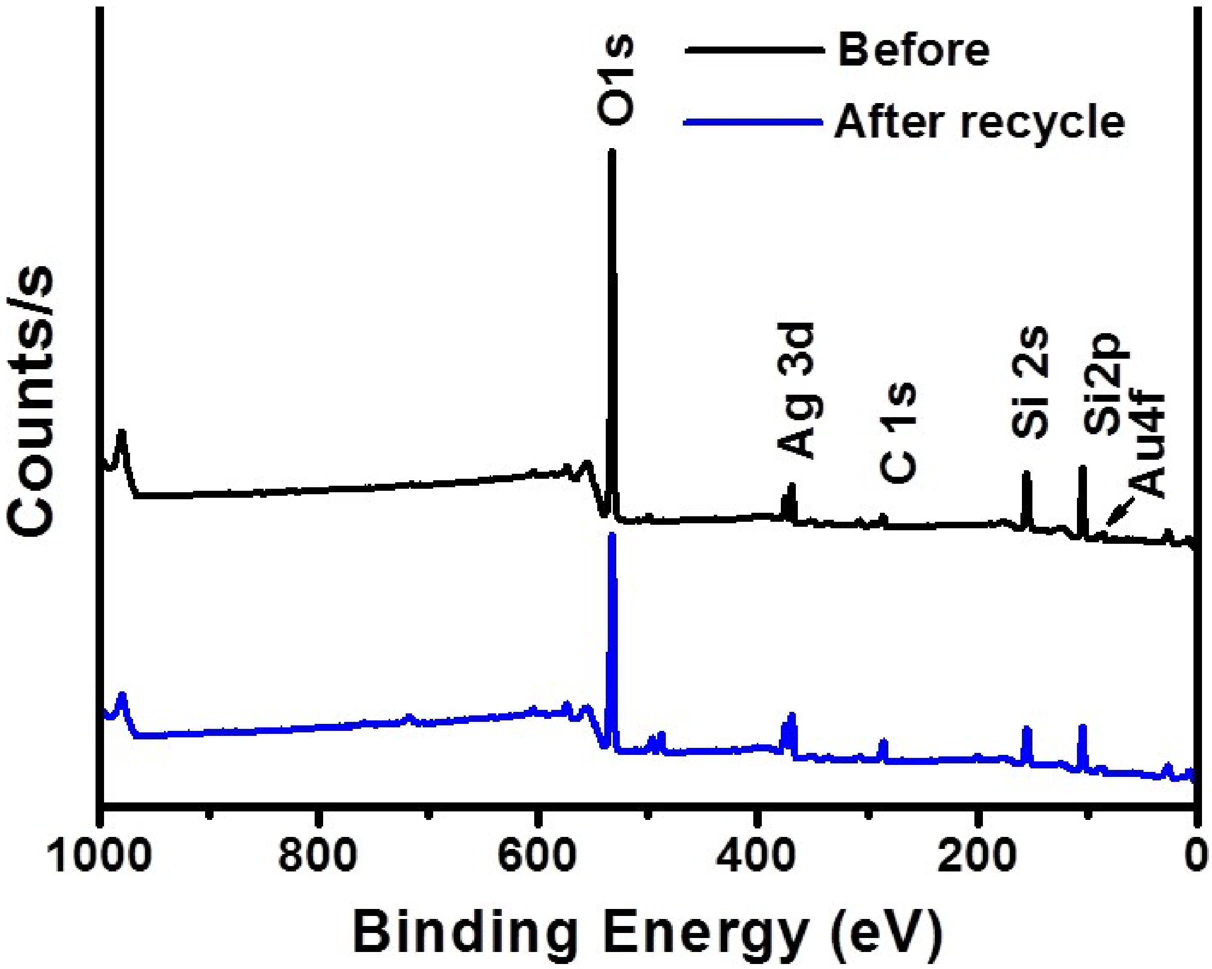

Figure S1 XPS survey spectra of SiO_2@Au-Ag NCs before and after catalysis

^{1}H NMR and ^{13}C NMR data and spectra of compounds 3a-3d

***N*-Phenylacetamide (3a):** ^{1}H NMR (600 MHz, $CDCl_3$) δ 8.00 (s, 1H), 7.49 (d, J = 7.9 Hz, 2H), 7.27 (t, J = 7.9 Hz, 2H), 7.07 (t, J = 7.4 Hz, 1H), 2.12 (s, 3H); ^{13}C NMR (150 MHz, $CDCl_3$) δ 168.8, 137.9, 128.8, 124.2, 120.0, 24.3.

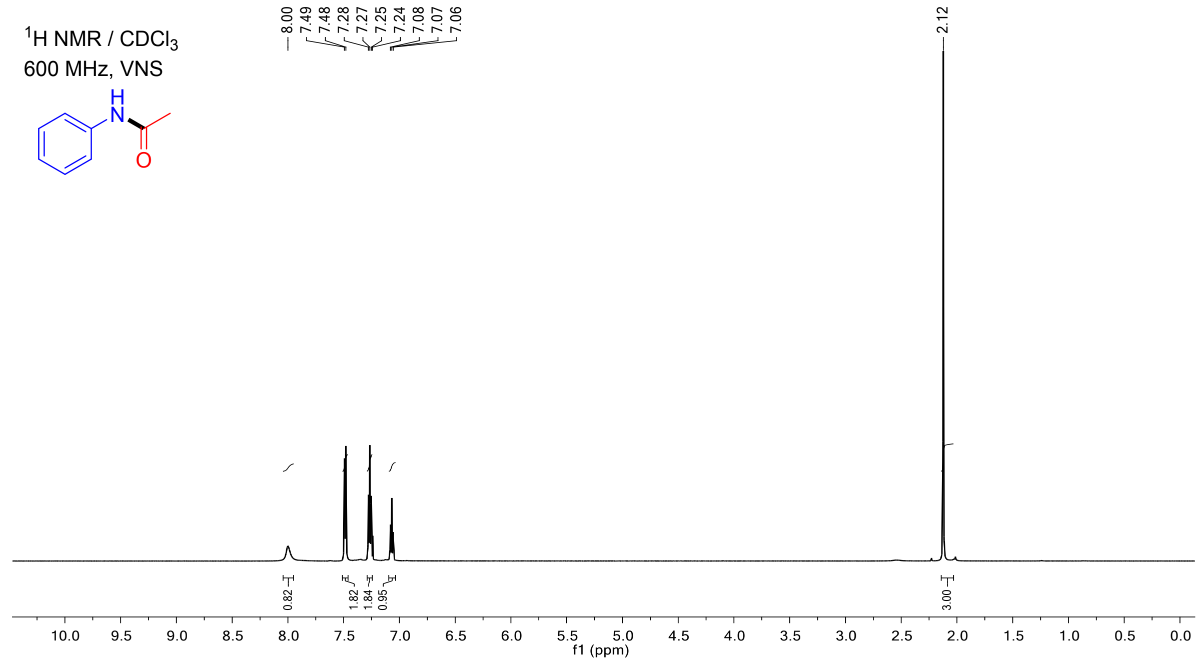

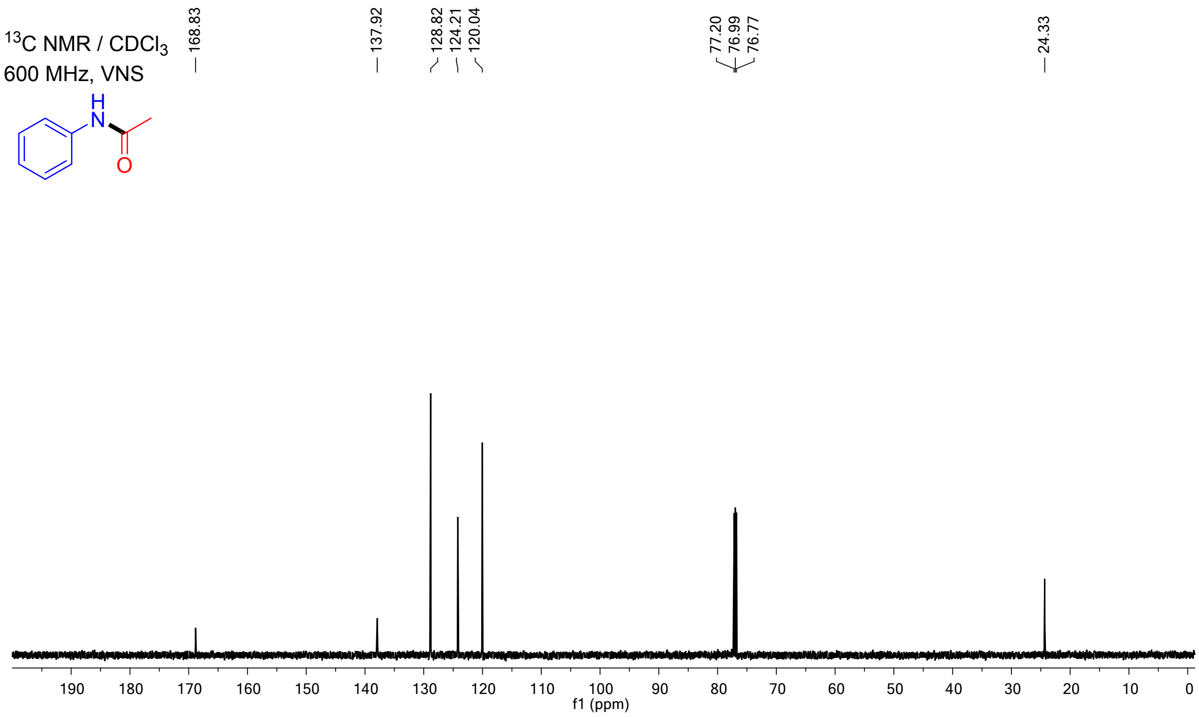

***N*-(3-chlorophenyl)acetamide (3b):** ^{1}H NMR (600 MHzCDCl$_3$) δ 8.27 (s, 1H), 7.62 (s, 1H), 7.31 (d, J = 8.0 Hz, 1H), 7.16 (t, J = 8.1 Hz, 1H), 7.03 (d, J = 7.8 Hz, 1H), 2.14 (s, 3H); ^{13}C NMR (150 MHz, CDCl$_3$) δ 169.2, 139.1, 134.4, 129.8, 124.2, 120.1, 118.0, 24.4.

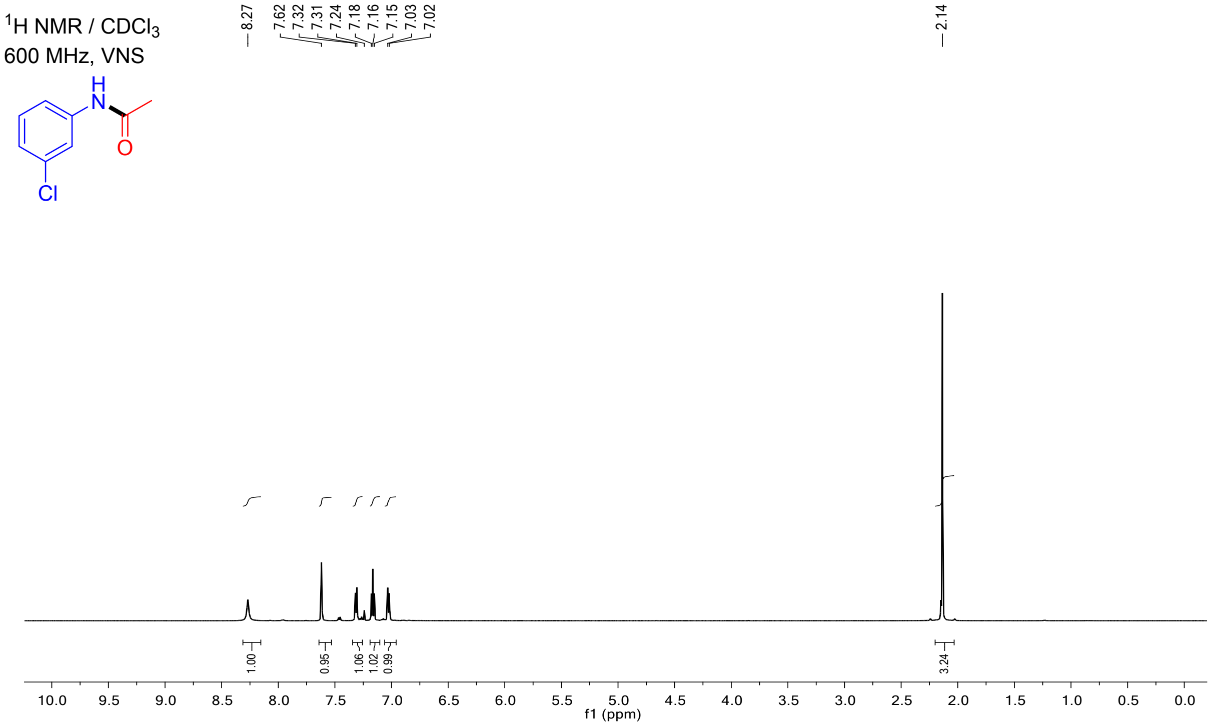

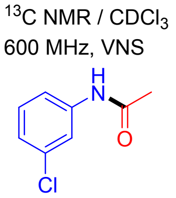

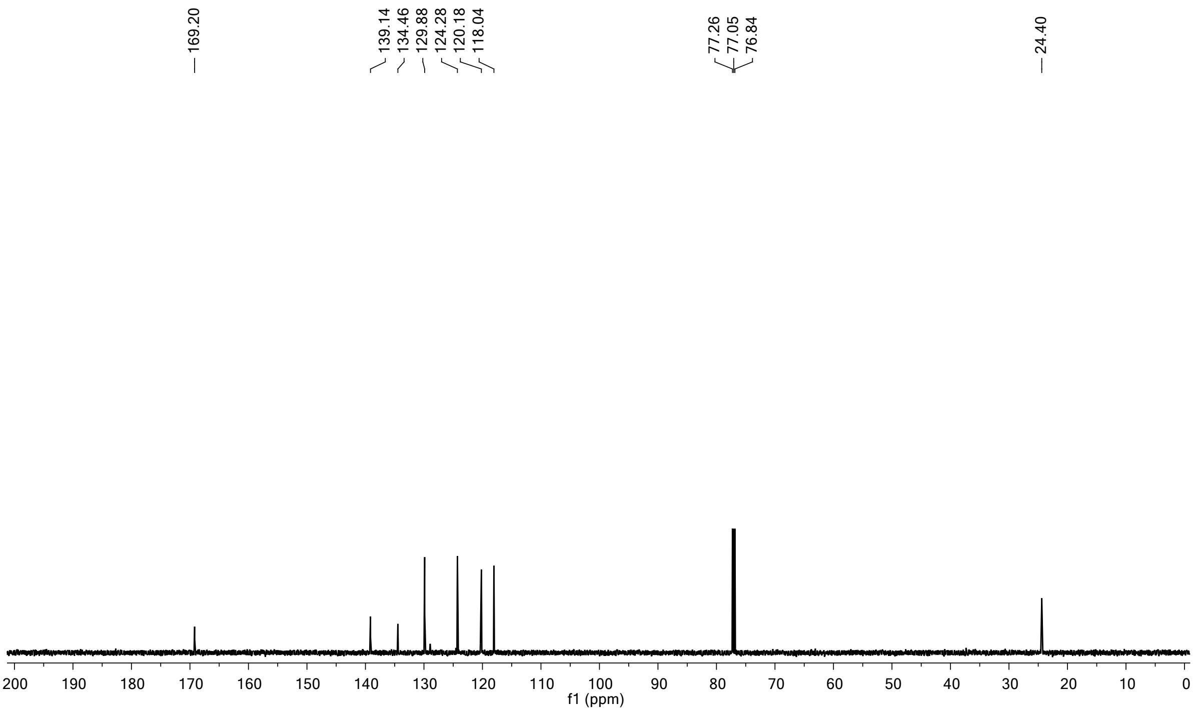

***N*-(4-Methoxyphenyl)acetamide (3c):** ^{1}H NMR (600 MHz, $CDCl_3$) δ 8.07 (s, 1H), 7.37 – 7.33 (m, 2H), 6.81 – 6.73 (m, 2H), 3.73 (s, 3H), 2.07 (s, 3H); ^{13}C NMR (150 MHz, $CDCl_3$) δ 168.8, 156.3, 131.1, 122.1, 113.9, 55.4, 24.0.

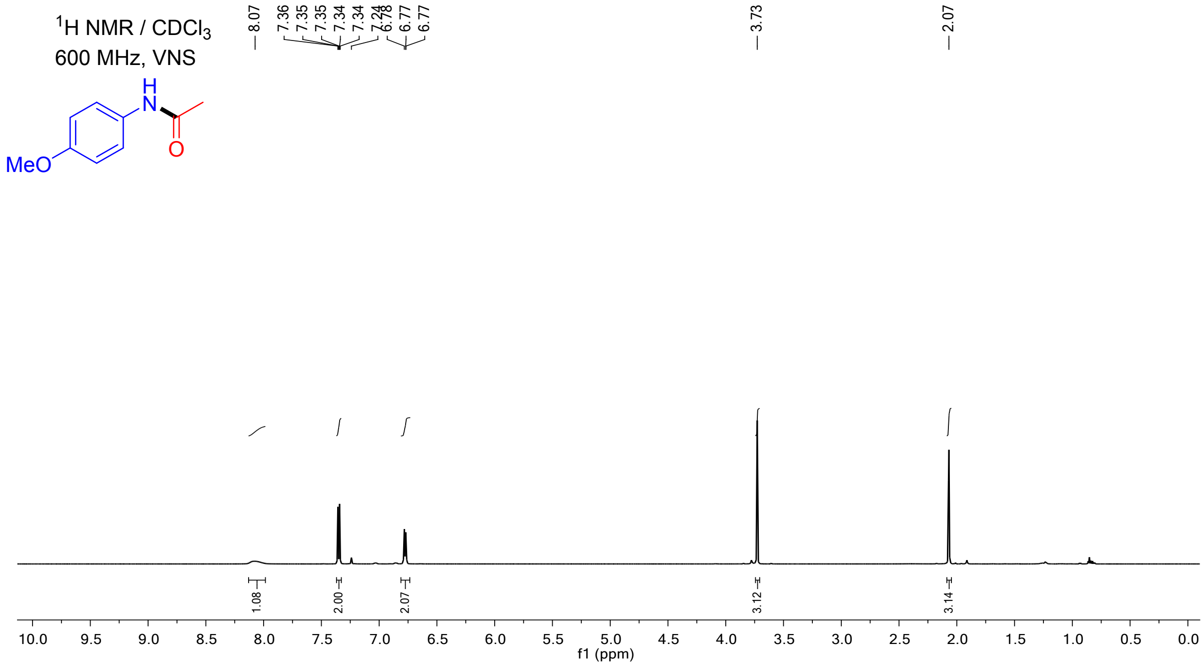

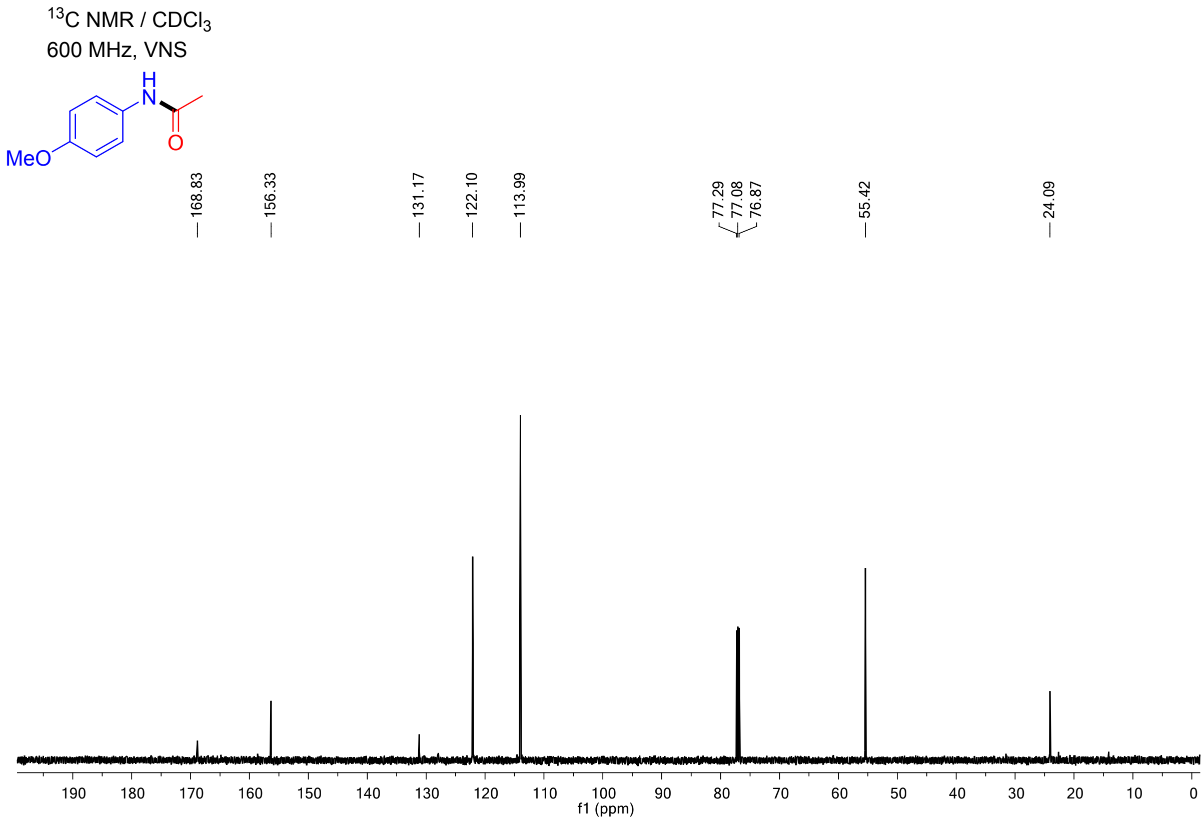

2,2,2-trifluoro-*N*-phenylacetamide (3d): ^{1}H NMR (600 MHz, CDCl$_3$) δ 8.13 (s, 1H), 7.59 – 7.55 (m, 2H), 7.39 (dd, J = 10.8, 5.1 Hz, 2H), 7.26 (dd, J = 11.7, 4.3 Hz, 1H); ^{13}C NMR (150 MHz, CDCl$_3$) δ 159.6, 154.9 (q, J = 37.5), 135.0, 129.2, 126.3, 120.6, 115.7 (q, J = 286.5 Hz).

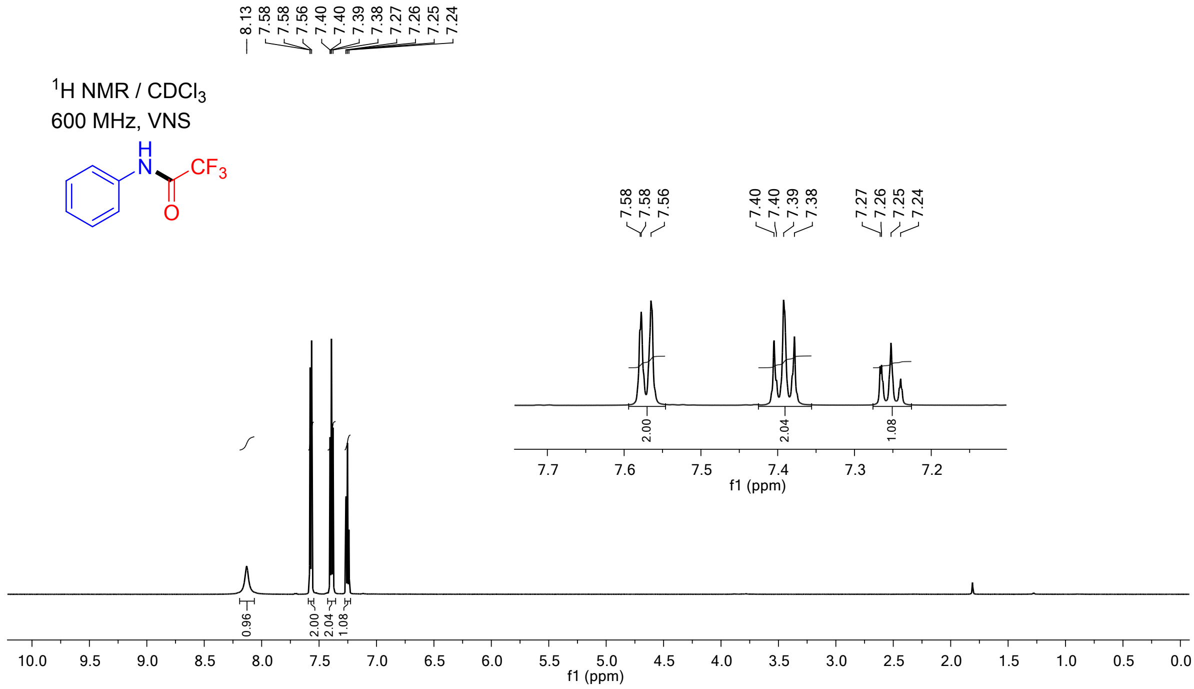

¹H NMR / CDCl3
600 MHz, VNS
CF3
8.13
7.58
7.58
7.56
7.40
7.40
7.39
7.38
7.27
7.26
7.25
7.24
0.96
2.00
2.04
1.08
f1 (ppm)

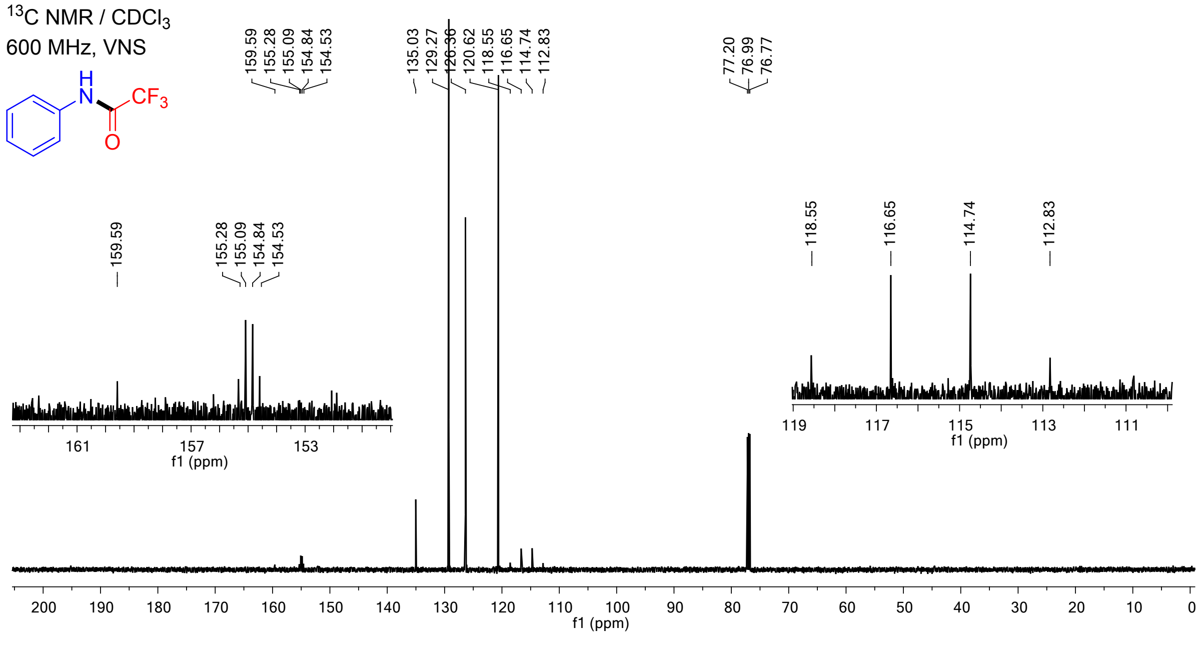

¹³C NMR / CDCl3
600 MHz, VNS
CF3
159.59
155.28
155.09
154.84
154.53
135.03
129.27
126.36
120.62
118.55
116.65
114.74
112.83
77.20
76.99
76.77
f1 (ppm)
